# Winner-loser effects improve social network efficiency between competitors with equal resource holding power

**DOI:** 10.1038/s41598-023-41225-y

**Published:** 2023-09-02

**Authors:** M. Hermanussen, M. Dammhahn, C. Scheffler, D. Groth

**Affiliations:** 1 Eckernförde-Altenhof, Germany; 2 Behavioural Biology, University of Münster, Munster, Germany; 3 Institute of Biochemistry and Biology, Human Biology, University of Potsdam, Potsdam, Germany; 4 Institute of Biochemistry and Biology, Bioinformatics, University of Potsdam, Potsdam, Germany

**Keywords:** Ecological networks, Evolutionary ecology, Social evolution

## Abstract

Animal societies are structured of dominance hierarchy (DH). DH can be viewed as networks and analyzed by graph theory. We study the impact of state-dependent feedback (winner-loser effect) on the emergence of local dominance structures after pairwise contests between initially equal-ranking members (equal resource-holding-power, RHP) of small and large social groups. We simulated pairwise agonistic contests between individuals with and without a priori higher RHP by Monte-Carlo-method. Random pairwise contests between equal-ranking competitors result in random dominance structures (‘Null variant’) that are low in transitive triads and high in pass along triads; whereas state-dependent feedback (‘Winner-loser variant’) yields centralized ‘star’ structured DH that evolve from competitors with initially equal RHP and correspond to hierarchies that evolve from keystone individuals. Monte-Carlo simulated DH following state-dependent feedback show motif patterns very similar to those of a variety of natural DH, suggesting that state-dependent feedback plays a pivotal role in robust self-organizing phenomena that transcend the specifics of the individual. Self-organization based on state-dependent feedback leads to social structures that correspond to those resulting from pre-existing keystone individuals. As the efficiency of centralized social networks benefits both, the individual and the group, centralization of social networks appears to be an important evolutionary goal.

## Introduction

Conflict over limited resources is the driving force of natural selection^1^ and an important determinant of social structure. Whenever animals associate in temporarily stable formations of social rank order hierarchies frequently emerge. As a result, nearly all animal societies are structured by some type of dominance hierarchy^2^ where an individual’s position within the social hierarchy usually reflects admittance to food and reproductive success. Albeit fighting over dominance status is costly, stable dominance hierarchies reduce fitness costs of fights at the individual and group-level^3^.

Dominance hierarchies are often displayed as linear rankings with each individual occupying a distinct social rank that is only defeated by individuals of higher ranks, but they can also be much more complex and viewed as networks which are “determined by asymmetrical displays of threat and, especially, subordination^2^”. Yet, the mechanisms underlying the formation of hierarchical social networks are not well-understood. Thus, we became interested in basic mechanisms that contribute to the formation and the topology of hierarchical social networks.

Investigating social structures by network and graph theory has a long history^3^. Past studies of networks have typically focused on global topological properties, such as density, degree distribution, assortativeness and dissortativeness, and clustering of networks. Yet, these properties tell little about the local structures within a network^4^. Faust and Skvoretz^5^ summarized 42 social networks from humans, mammals, and birds. Comparing social structures across taxa, they concluded “that similarities among the networks are more due to the kind of relation than to the kind of animal”. More recently, Shizuka and McDonald^6^ applied network motif analysis on triad configurations in dominance hierarchies in 172 animal groups published in 113 studies on 86 taxa. The analysis of triads was shown to be a valid approach to examine the local topology of a network. Triads are subsets of three nodes within a network. Shizuka and McDonald stated that “triad motifs across dominance networks revealed general patterns in the structures of dominance hierarchies across virtually all animals” with no substantial differences among the taxonomic groups in the structure of dominance hierarchies^6^. In the analyzed animal groups, double-dominant (A dominates both B and C) and transitive triads (A dominates B and C, and B dominates C) occurred more frequently than expected, pass-along triads (A dominates B and B dominates C) less frequently than expected, and cycle triads (A dominates B, B dominates C, and in return, C dominates A) least of all. Shizuka and McDonald identified social dynamics as major determinants shaping the hierarchy structure of a group. In line with common notion, they highlighted the importance of ‘keystone individuals’ as a major driver of variation in dominance hierarchies across taxa. Triad motifs are ubiquitously found in networks from biochemistry, neurobiology, ecology, and engineering^7^.

Individuals vary in their ecological impact with different resource holding power (RHP). Yet, the terminology of individuals with a disproportionately small or large and irreplaceable effect on group dynamics is not unambiguous. In terms of Modlmeier and coworkers^7^ we distinguish between **‘**keystone individuals’ that are the product of pre-existing genetic elements (i.e., keystone-conferring genotypes) that evolve under Darwinian selection as the product of selective forces on the keystone genotype; and individuals with a ‘keystone role’ the latter being an experiential, state-dependent, or context-dependent phenomenon.

The assumption that some individuals act as keystone individuals with strong effects on social network formation is intuitive and prevailing, but has rarely been tested by Monte-Carlo simulation, and if, under different perspectives^8^. Therefore, we follow arguments of Maynard Smith & Price^3, 9^ and consider the struggling for dominance under aspects of the theory of games and modeled the evolvement of social networks with winners, losers, or with a draw by Monte-Carlo simulation. Our main aim was to illuminate whether dynamics of social hierarchy formation following pairwise agonistic contests with or without keystone individuals, i.e., single competitors with or without an a priori advantage, lead to the emergence of qualitatively similar social network structures.

Most theoretical game experiments focus on the functionality of networks. They model cooperation and promotors of cooperation among group members, such as the Prisoner’s Dilemma, the Snowdrift Game, the Stag-hunt Game and others^10^. The present study is restricted to considerations about the evolution of topological structure of dominance hierarchies and builds on previous theoretical modelling work considering homogeny of RHP among group members as a “fundamental ingredient in the dynamics of group (that is, social) network formation^11^”. We studied the effects of contests between initially equal-ranking group members, and under the premise of random analyzed the consequences of state-dependent behavioral feedback (winner-loser effect)^12^. The winner-loser effect describes the probability to win or lose a fight depending on the outcome of previous interactions due to changes in the individual’s assessment of its own cost/benefit ratio of fighting^13, 14^. The introduction of co-evolutionary rules to evolutionary games implies that besides the evolution of strategies, other properties may simultaneously be subject to evolution as well. A large body of literature on how state-dependent feedbacks might shape social networks has been summarized by Perc and Szolnoki^10^.

By means of Monte-Carlo technique we simulated the effect of serial pairwise contests (everyone against everyone) yielding winners, losers, or a draw on the topology of the resulting dominance hierarchy. We then compared the new hierarchy to random hierarchies and applied network and graph theory. We hypothesized that (1) state-dependent feedback (winner-loser effect) leads to network centralization with more double dominant and transitive triads and less cyclic triads than expected, and that (2) the effect of state-dependent feedback on equal-ranking competitors equals that of single keystone individuals on the evolution of dominance hierarchies. Initially, we studied small networks of 12 individuals. Yet, given the importance of network size^15^, we also extended the model and studied networks with up to 20 × 20 individuals.

## Material and methods

### Networks

Networks consist of distinct elements or actors (the competing individuals) represented by *nodes* and connections between nodes that are called *edges*. Networks differ by size, by density, they may cluster, and centrality indices produce rankings for identifying the most important nodes. Global reaching centrality is a hierarchy measure of complex networks^16^. The path lengths between nodes depict how integrated different parts of a network are with global efficiency being defined as the average shortest path length over all pairs of nodes^17, 18^. Efficiency means efficiency of information transport and determines network functionality, but also affects its resilience to failure or attacks^19^ and characterizes global network features. We however, focused on local network structures with particular emphasis on network building blocks^20^ and the prevalence of triads, i.e. network subgraphs consisting of three connected nodes. Because of our interest in local structures, we studied both small networks where everyone interacts with everyone and large networks where everyone is only able to interact with closest neighbors.

### Monte-Carlo simulation

We simulated seasons of pairwise agonistic contests. Seasons consist of n*(n-1)/2 contests (“everyone against everyone”), i.e., in the case of 12 competitors, one season comprises 66 contests. The number of all possible contests grows squared with the number of competitors. We therefore changed the "everyone against everyone" rule to “everyone against closest neighbors" in the large networks. We defined artificial rectangular landscapes similar to fishermen networks with a standard edge length of 1.0 ± ε (with ε = 0.1) between neighboring nodes and assigned each competitor a position within this network. The probability of contest between neighboring competitors was defined by a Gompertz’ function

$$y+1={a*e}^{-{b*e}^{-c*x}}$$

The function defines the probability of a contest by the spatial distance (*x*) between two neighbors. We set a = − 1 in order to reverse the function and chose a wide range of values for the variables *b* and *c*. *b* sets the displacement along the *x*-axis (translates the graph to the left or right, i.e., determines the average distance at which the probability of a contest between two competitors is halved). *c* sets the y-scaling, i.e., the gradient from high to low probability for a contest.

All calculations were performed with R^21, 22^. The R code for the simulation is freely available at https://github.com/mittelmark/hanna.,

### The “Resource Token Game”

We assigned each competing individual a certain number of tokens representing its RHP and simulated contests between two competitors (e.g., A contra B, B contra C). Fighting ability depends on RHP: the higher the number of tokens the higher its probability of winning the contest. Initially all competitors were equal-ranking, they started with the same number of tokens, i.e., were “homogenous” in their initial RHP. The game implies that both competitors put their tokens into an urn from which two tokens are randomly withdrawn. The outcome of the contest is as follows: AA (both tokens from individual A) indicates: A is dominant, BB indicates: B is dominant, AB or BA indicates a drawn. An individual’s chance of winning is proportional to its relative number of tokens in the urn. The “Resource Token Game” can be adopted within a broad frame of applications including economist's logic of resource agglomeration and rich-get-richer dynamics. The game exhibits robust self-organizing phenomena that transcend the specifics of the individual similar to what has been described as preferential attachment by Barabási and Albert^23^. Individuals who lost all tokens can no longer participate in the game.

First, we studied ‘Null variant’ games where equal-ranking (homogeny of initial RHP) competitors played with ¼ chance of winning, ¼ chance of losing, and ½ chance of a draw. The outcomes of ‘Null variant’ games are randomly wired small-world^15^ winner-loser networks. This is different when introducing state-dependent feedback (‘winner-loser variant’ games). State-dependent feedback takes the outcome of previous contests into account. Previous winners start a new game with plus one token, previous losers start with minus one token. Winners become “rich” (winner effect), losers become poor (loser effect)^24^. In ‘Memory5 variant’ games competitors only “remember” the last five contests, in full ‘Winner-loser variant’ games competitors “remember” all previous contests. In ‘Keystone variant’ games one (the keystone) individual starts with an initially greater number of tokens and tends to win the following games. ‘Keystone variant’ games closely resemble the ‘Winner-loser variant’ games. We simulated one, five, 10 and 30 seasons.

The “Resource Token Game” may appear trivial at first view, but as everyone fights against everyone, the outcome is a highly dynamic network of winners and losers that self-organizes with increasing number of seasons. The patterns of self-organization are largely independent of the size of the network. To retain visibility of the Figures, we only show winner-loser dyads and disregard the drawn. More sophisticated variants of the “Resource Token Game” separating winner effects and loser effects^14^, yielded very similar results and thus, were not further considered.

### Network analysis

We simulated networks resulting from pairwise contests yielding winners, losers, or draws. We started with 12 competitors (everyone against everyone), and extended the analysis to up to 20 × 20 competitors within an artificial landscape and varying probability of contests according to the spatial distance between competitors. We studied average shortest path length over all pairs of nodes^17, 18^ to estimate network efficiency, and applied network motif analysis considering five triad motifs^6^:

- dd: double dominant (A dominates B and C).
- ds: double subordinate (“servants of two lords”).
- pa: pass-along (A dominates B and B dominates C).
- tr: transitive (A dominates B and C, and B dominates C).
- cy: cycles (A dominates B, B dominates C, and in return, C dominates A).

## Results

The present study shows the emergence of dominance structures after pairwise contests between initially equal-ranking members of small and large social groups. All simulations were tested against ‘Null variant’ games (no state dependent feedback).

### Small networks: ‘everyone against everyone’

**Figure 1:**
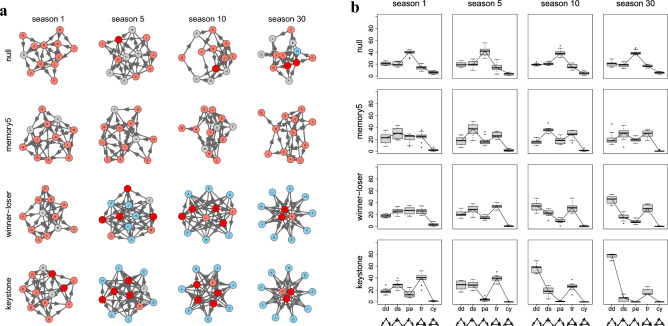
(**a**) Representative examples of four small simulated networks evolving from 1, 5, 10, and 30 seasons of 66 pairwise contests between 12 competitors (‘everyone against everyone’). Arrows indicate winners and losers of the last contests (winner → loser). Colors indicate accumulated number of tokens (red: > 20 tokens, pink: 10–20 tokens, gray: 3–9 tokens, light blue 0–1 token). ‘Null variant’: Each competitor starts with the same number of tokens (“homogeny” of the initial resource-holding-power). Odds of winning are (¼), losing (¼) or drawing (½). Outcomes of previous contests are not taken into account. ‘Memory5 variant’: Competitors “remember” the last 5 contests. ‘Winner-loser variant’. Competitors “remember” the outcome of all previous contests. Winners steadily accumulate, whereas losers progressively lose tokens. These networks turn into centralized ‘star’ networks with one or a few centrally situated individuals. ‘Keystone variant’: Keystone network with at least one individual with a priori higher resource holding power. In this simulation, the individual remained in the group of three central individuals. (**b**) Triad motif patterns (mean values, 2nd and 3rd quartiles, and 95% confidence intervals) in 10 repetitions. ‘Null variant’ games remain random, whereas ‘Winner-loser variant’ and ‘Keystone variant’ games quickly centralize. *dd* double dominant, *ds* double subordinate, *pa* pass-along, *tr* transitive, *cy* cyclic triads.

Figure 1a and b exemplifies the evolution of representative dominance structures after pairwise ‘everyone against everyone’ contests among 12 competitors. The first rows illustrate the ‘Null variants’, the second rows the ‘Memory5 variants’, the third rows the ‘Winner-loser variants’, and at bottom, the ‘Keystone variants’. Figure 1a depicts the logical graph structures based on the distances given by the shortest undirected paths between neighboring nodes with arrows indicating winners and losers of the last contests. State-dependent feedback results in substantial centralization of the dominance structure.

‘Null variant’ networks have generally short average shortest path length over all pairs of nodes^15^. Ten repetitions yielded average shortest path length of 1.6 (SD 0.1) standard distances between competitors that remained unaltered over 30 seasons. ‘Winner-loser variant’ and ‘Keystone variant’ networks had slightly shorter average shortest path length [mean 1.3 (SD 0.1) standard distances], but significantly differ in network motif patterns.

Figure 1b illustrates triad patterns (mean values, 2nd and 3rd quartiles, and 95% confidence intervals) in 10 repetitions. Triad patterns of ‘Null variant’ games maintain their initial random structure with many pass along and a few cycle triads during 30 seasons, whereas ‘Winner-loser variant’ and ‘Keystone variant’ games quickly centralize. ‘Memory5 variant’ games show an intermediate pattern. Whereas pass-along triad are the most common triads in the ‘Null variant’, double dominant and transitive triads are significantly overrepresented in the ‘Winner-loser’ and in the ‘Keystone variant’ games. Cyclic triads almost completely disappear in state dependent feedback (*p* < 0.05). In ‘Winner-loser variant’ games any individual may achieve a keystone position, in ‘Keystone variant’ games, it is usually, but not always, the original keystone individual that remains in its keystone position through the game.

### Small networks: ‘everyone against closest neighbors’

**Figure 2:**
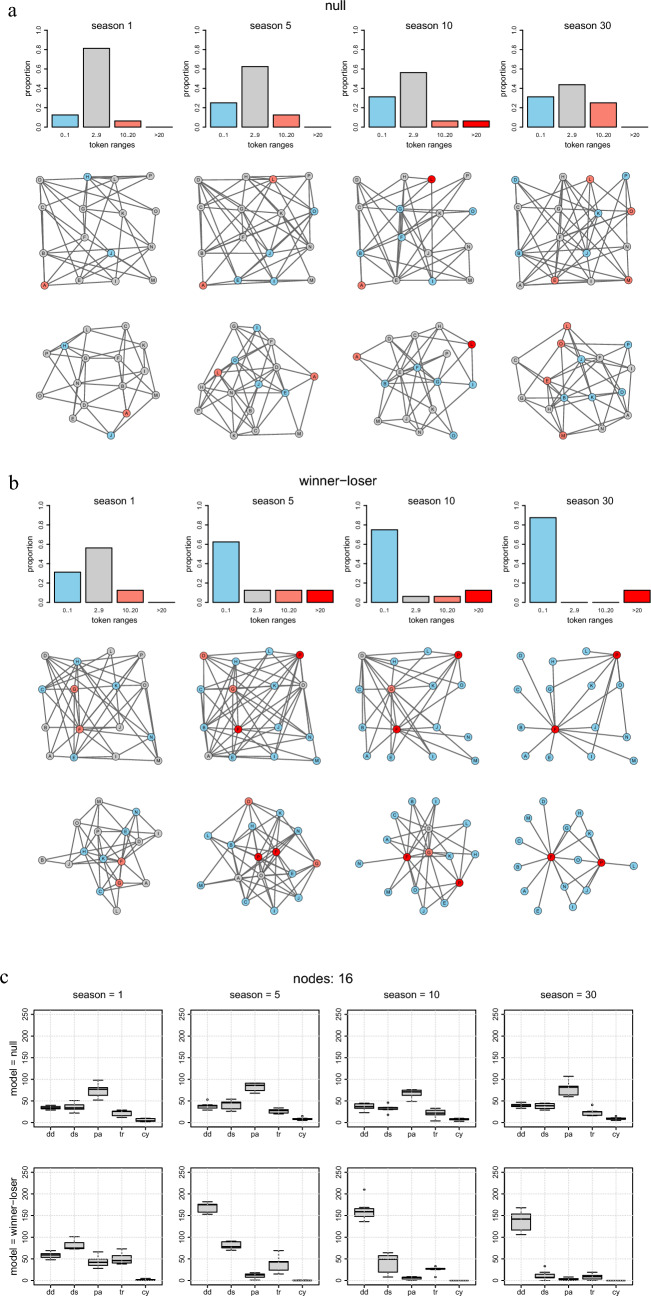
Representative examples of 4 × 4 simulated dominance structure evolving from 1, 5, 10, and 30 seasons of pairwise contests (‘everyone against closest neighbors’). Gompertz variables *a* = − 1, *b* = 15, and *c* = 1.0). (**a**) ‘Null variant’ network, and (**b**) ‘Winner-loser variant’ network. Upper rows: Histogram of token distribution, Middle rows: Spatial distribution of winners and losers on a rectangular 4 × 4 landscape, Bottom rows: Logical graph structures based on the distances given by the shortest undirected paths between the nodes. (**c**) Triad motif patterns (mean values, 2nd and 3rd quartiles, and 95% confidence intervals) from 10 repetitions.

Figure 2a and b exemplifies the evolution of small representative dominance structures after pairwise ‘everyone against closest neighbors’ contests among 16 competitors (Gompertz parameters *a* = − 1, *b* = 15, *c* = 1.0). ‘Memory5 variants’ exhibited intermediate patterns (not shown); ‘Keystone variants’ (not shown) remained indistinguishable from ‘Winner-loser variants’. The distribution of tokens is depicted in the upper rows and the spatial distribution of winners and losers in the center rows. The logical graph structures based on the distances are given by the shortest undirected paths between the nodes in the bottom rows. Due to the randomness of which neighbor competes with whom, the distribution of tokens widens with increasing season also in the ‘Null variant’ games. The star-shape of the ‘Winner-loser variant’ is obvious with few wealthy winners (red bars) and many losers (blue bars), though slightly less distinctive than in the ‘everyone against everyone’ game.

Triad motif patterns derived from 10 repetitions of ‘everyone against closest neighbors’ are given in Fig. 2c. The patterns are similar to those emerging from ‘everyone against everyone’ contests in small groups, with significant overrepresentation of double dominant and transitive triads (*p* < 0.05).

### Large networks: ‘everyone against closest neighbors’

**Figure 3:**
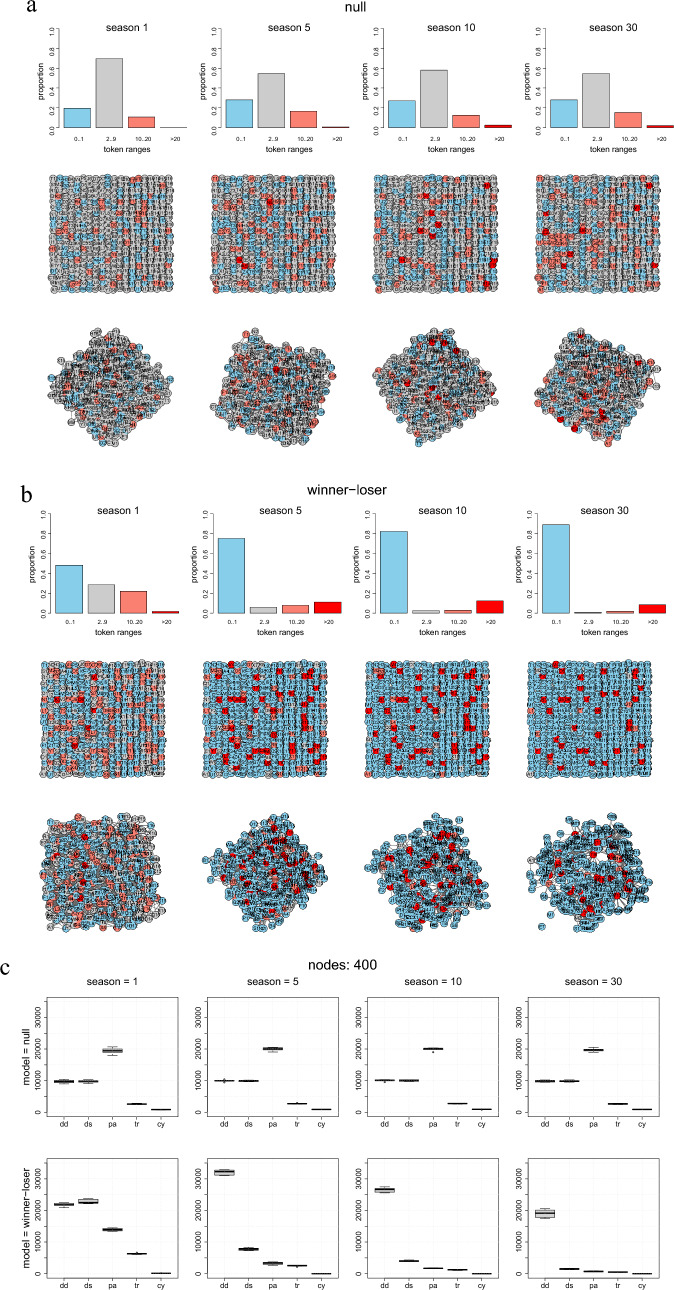
Representative examples of a 20 × 20 simulated dominance structure evolving from 1, 5, 10, and 30 seasons of pairwise contests (‘everyone against closest neighbors’). Gompertz variables *a* = − 1, *b* = 15, and *c* = 1.0). (**a**) ‘Null variant’ network, and (**b**) ‘Winner-loser variant’ network, Upper rows: Histogram of token distribution, Middle rows: Spatial distribution of winners and losers on a rectangular  20 × 20 landscape, Bottom rows: Logical graph structures based on the distances given by the shortest undirected paths between the nodes. (**c**) Triad motif patterns (mean values, 2nd and 3rd quartiles, and 95% confidence intervals) from 10 repetitions.

Figure 3a and b exemplifies the evolution of dominance structures within a 20 × 20 network following pairwise ‘everyone against closest neighbors’ contests after 1, 5, 10, and 30 seasons (Gompertz parameters *a* = − 1, *b* = 15, *c* = 1.0). With state dependent feedback, the distributions of tokens are very asymmetric with few winners (red bars) and a large number of losers (blue bars).

Figure 3c depicts triad motif patterns derived from 10 repetitions. Triad patterns found in large networks differ from small ones in that transitive triads disappear, whereas double dominant triads almost exclusively displace the formation of the other triads.

For reasons of practicability, we restricted our analysis to exemplary results for (1) *a* = − 1, *b* = 15, and *c* = 1.5 and (2)* a* = − 1, *b* = 15, and *c* = 1.0. Modifying the probability of a contest between two competitors does, but does not substantially, modulate the effects of state dependent feedback on local network structures. With *c* = 1.0, the group of potential neighboring competitors in a 20 × 20 rectangular network spans about three standard distances and is 31.0 (SD 7.6) for each individual. With *c* = 1.5 the group of potential neighboring competitors in the same network is 14.2 (SD 3.6). Large numbers of potential neighboring competitors yield dominance network with few, but very rich keystone positions; small numbers of potential neighboring competitors yield many, but less wealthy keystone positions.

The number of competitors also depends on network size, as the proportion of peripheral nodes (with fewer numbers of neighbors at the verge) relative to all nodes decreases with size. Yet, this effect was small and without substantial impact on the evolution of star-shaped hierarchical structures (data not shown).

## Discussion

Nearly all animal societies are structured by some type of dominance hierarchy^2^. Shizuka and McDonald^6^ applied network motif analysis to compare the structures of dominance networks from data published over the past 80 years. They showed that the overall patterns of dominance relations did not vary in any systematic way across taxa, study settings (captive or wild) or group size, but were strikingly similar across disparate animal groups. Nearly all groups exhibited high frequencies of double-dominant and transitive triads, whereas pass-along triads were rare, and cycles very rare. Similar phenomena have been observed in regulation networks of direct transcriptional interactions in *Escherichia coli*^25^. Transitive triads (feedforward loops) are numerously found in diverse biological systems at rates that surpass the mean number of appearances in randomized networks by the factor ten^20^, whereas cycle and pass along structures seem to be significantly underrepresented.

In this study we show that in depth considerations including a “multi-dimensional view of social structure that incorporates the dynamics of unobserved or unobservable social interactions, as well as the temporal dynamics of how hierarchies emerge …”^6^, may possibly not be required. Based on Monte-Carlo simulation of social hierarchy formation, we suggest that complex dominance hierarchies can randomly evolve from pairwise agonistic contests when allowing for state-dependent feedback.

Competitions between individuals with an a priori higher resource holding power (RHP) (‘keystone individuals’) usually result in social network centralization. This is an autonomous statistical phenomenon and reflects the Darwinian concept of Natural Selection preserving favorable variations, survival of “the vigorous, the healthy, and the happy” and rejecting injurious variations^1^. We show that the same occurs between equal-ranking individuals in a state-dependent feedback system (winner-loser effect)^12^ where a successful competitor can increase his odds of winning in future contests and establish a future ‘keystone role’^7^ simply for statistical reasons. Our findings support previous game theoretical modelling work that considered homogeny of RHP as a fundamental ingredient in the dynamics of network formation^11^. Winner-loser effects mildly reduce the average shortest path length over all pairs of nodes in small random networks, but they do not seem to significantly contribute to global network efficiency^17, 18^. Random networks of any size show “small-world” features with “six degrees of separation”^15, 26^, and thus, are already quite “efficient” in the sense of network theory. Winner-loser effects as simulated in this study, do not further reduce average path length over all pairs of nodes, but lead on the local level to substantial centralization. Winner-loser effects significantly augment the number of double dominant and transitive triads in networks where everyone struggles with everyone, and in small networks where everyone struggles with its closest neighbors. The present simulation shows that winner-loser effects in larger networks only raise the number of double dominant triads, but preliminary data suggest that the number of transitive triads also increases when “socializing” the instructions by donating one or two tokens to the poorest, so that they may continue in the game (work in preparation).

Triads are considered the simple building blocks of which complex networks are composed^20^. Triads are widely used for describing biological^25^, social^27, 28^ and even trade networks^4^. The present study shows that binary contests between equal-ranking competitors in a Monte-Carlo setting that allows for state-dependent feedback, create dominance structures that are strikingly similar to the structure of social networks observed in biology^6^.

Double dominant and transitive pathways favor, while pass along (“Chinese whispers”) and especially circular pathways tend to impair the flow of information in top-down command structures. The present study thus, suggests that the fundamental evolutionary concept of "struggle for survival" is not so much one that selects for “the vigorous, the healthy, and the happy”^1^, but creates social structures that raise the efficiency of information transfer within social groups through the means of network mathematics.

The present results confirm our first hypothesis in that state-dependent feedback (winner-loser effect) leads to network centralization with overrepresentation of double dominant and transitive triads, and with fewer cyclic triads than expected. The strength of the centralizing effect depends on the magnitude of “remembering” previous contests. The longer the memory, the more the resulting network structures become indistinguishable from those that emerge in the presence of single keystone individuals with an a priori higher RHP.

The results highlight the importance of feedback and also confirm our second hypothesis. Random dual contests between equal-ranking individuals are unable to substantially structure social networks. But introducing feedback enables the centralization of dominance structure similar or even equal to that seen in the presence of *priori* higher RHP ‘keystone individuals’. State-dependent feedback mimics the “natural selection of the vigorous, the healthy, and the happy”^1^ in that it favors and selects for greater resource holding power. In contrast to natural selection however, the “happy” individuals had never been different from the others in the first place. i.e., selection occurs solely on the basis of “memorizing” a previous state that was acquired by random.

We consider the similarity between triad motif structures found in Monte-Carlo simulations and those found in a broad variety of empirically described dominance hierarchies^6^ and the coincidences with previous mathematical considerations on self-organizing dominance structures in animal societies^29^ as strong evidence for a fundamental biological role of the combination of random selection and state-dependent feedback. This combination appears crucial for the evolution of efficient social networks between competitors even if they had previously never differed in resource holding power. Winner-loser effects cause network centralization. The formation of centralized social structures appears beneficial not so much because it shortens the average path lengths, but because it facilitates the formation of top-down star-like structures. Top-down command structures with a single or a few influential individuals in the center surrounded by many dependents have been shown to facilitate information transport within the group and are—particularly in modern autocratic societies—vividly discussed as efficient control and management regimes^30, 31^, and may significantly foster efficiency and functionality of any biological network^19^.

State-dependent feedback plays an important role for the understanding of non-random frequency patterns of biological network motifs. State-dependent feedback prioritizes double dominant and—in the case of small networks—also transitive triads. Both motifs are widely observed in natural settings, they appear to be important determinants of social structure, and they support short chains of command. Yet it must be kept in mind that human societies are by far more complex, and that formal hierarchical structures detected in random simulations do not map into informal social networks.

Monte-Carlo simulations of social network evolution are highly simplified approaches to understanding the mechanisms that control the evolution of real networks. This study is limited to understanding the effect of pairwise competition between competitors with equal RHP in the presence of state-dependent feedback in a rigid computer model. We restricted our approach to analysing small networks consisting of 12 and 16 competitors, and a few larger networks consisting of up to 400 competitors. Any human society is by far more complicated than such an approach that simply focuses on local specifics and manages the redistribution of tokens between neighboring competitors. Yet, triad analysis has even been suggested to provide policy tools for areas ranging from national security to environmental management^4, 32^. Future work should investigate the impact of more complex models on the evolution of hierarchical structures within random networks of larger size and higher clustering^15^.

## Conclusion

The present study shows the emergence of dominance hierarchies after pairwise contests between initially equal-ranking members of small and large social groups. Monte-Carlo simulated dominance hierarchies following state-dependent feedback show motif patterns very similar to those of a variety of natural dominance hierarchies, suggesting that state-dependent feedback plays a pivotal role in robust self-organizing phenomena that transcend the specifics of the individual. Self-organization based on state-dependent feedback leads to social structures that correspond to those resulting from pre-existing key individuals. As the efficiency of centralized social networks benefits both, the individual and the group, centralization of social networks appears to be an important evolutionary goal. Command structures with a single or a few influential individuals in the center surrounded by many dependents are ubiquitously found also in modern human societies.

## Data Availability

The datasets simulated during the current study are free available https://github.com/mittelmark/hanna.
